# A web-based interface to calculate phonological neighborhood density for words and nonwords in Modern Standard Arabic

**DOI:** 10.3758/s13428-021-01713-3

**Published:** 2022-01-10

**Authors:** Faisal Aljasser, Michael S. Vitevitch

**Affiliations:** 1 Department of English Language and Translation, College of Arabic Language and Social Studies, Qassim University, Buraydah, 52571 Saudi Arabia; 2 Spoken Language Laboratory, Department of Psychology, University of Kansas, 1415 Jayhawk Blvd, Lawrence, KS 66045 USA

**Keywords:** Phonological neighborhood density, Modern Standard Arabic, Online calculator

## Abstract

The availability of online databases (e.g., Balota et al., 2007) and calculators (e.g., Storkel & Hoover, 2010) has contributed to an increase in psycholinguistic-related research, to the development of evidence-based treatments in clinical settings, and to scientifically supported training programs in the language classroom. The benefit of online language resources is limited by the fact that the majority of such resources provide information only for the English language (Vitevitch, Chan & Goldstein, 2014). To address the lack of diversity in these resources for languages that differ phonologically and morphologically from English, the present article describes an online database to compute phonological neighborhood density (i.e., the number of words that sound similar to a given word) for words and nonwords in Modern Standard Arabic (MSA). A full description of how the calculator can be used is provided. It can be freely accessed at https://calculator.ku.edu/density/about.

Phonological neighborhood density (PND) refers to the number of words that sound similar to a given word. The phonological neighborhood of a given word is said to be dense when it has many phonologically similar words or neighbors. However, when the word has few neighbors, it is said to have a sparse phonological neighborhood.

A simple and widely used method (Greenberg & Jenkins 1964, Landauer & Streeter 1973) to operationally define the phonological similarity of two words is to use a variant of the Hamming or Levenshtein distance (see Luce & Pisoni, 1998 for other ways to operationally define phonological similarity). According to this method, two words are considered phonologically similar if the addition, deletion, or substitution of a phoneme in any position of one word produces the other word. For example, the English words *spat* /spæt/, *at* /_æt/, and *cat* /kæt/ are all neighbors of the word *pat* /pæt/ based on the addition, deletion, or substitution of a phoneme, respectively.

Although there has been some research on PND effects in other languages (e.g., *French*: Dufour & Frauenfelder, 2010; *Spanish*: Vitevitch & Rodriguez, 2005), most research has been conducted in English. These include studies of PND effects on word learning (e.g., Storkel, Armbrüster, & Hogan, 2006), speech disorders (e.g., Anderson, 2007), and short term memory (e.g., Roodenrys, Hulme, Lethbridge, Hinton, & Nimmo, 2002).

In spoken language processing of English, PND has been shown to affect speech production and speech recognition differently. For a review of how phonological neighborhoods influence spoken word perception and production, see Vitevitch and Luce (2016). Findings in speech production studies in English point to facilitative effects of PND (e.g., Vitevitch, 2002b; Vitevitch & Sommers, 2003) with words with dense neighborhoods being produced more quickly than words with sparse neighborhoods. It has been argued (Rapp & Goldrick, 2000) that such effects support interactive models of speech production (e.g., Dell & Gordon, 2003) instead of feed-forward models of speech production (e.g., Levelt, Roelofs & Meyer, 1999).

However, speech recognition studies in English have shown effects of lexical competition, with words with dense neighborhoods being responded to more slowly than words with sparse neighborhoods. For example, English words from dense neighborhoods are recognized more slowly and less accurately than words from sparse neighborhoods (e.g., Luce & Pisoni, 1998; Vitevitch & Luce, 1998) and result in more speech perception errors known as slips of the ear (Vitevitch, 2002a). Models of spoken word recognition have accounted for such effects by positing competition among phonologically similar word forms, as in the Neighborhood Activation Model (NAM; Luce & Pisoni, 1998), or by positing an inhibitory mechanism among word-forms, as in the TRACE model (McCelland & Elman, 1986).

Although effects of PND in speech perception, speech production, and other cognitive processes have been studied in English for some time, it is important to examine how phonologically similar words influence language-related processes in other languages. Part of the reason to do so relates to the fact that different languages have different phoneme inventories, different phonotactic rules governing the sequencing of phonemes in words, differences in morphological productivity, and differences in a number of other parameters (Arbesman, Strogatz & Vitevitch, 2010b).

Indeed, the limited amount of research in other languages shows important differences in how phonologically similar words influence perception and production in Spanish compared to English. For example, Vitevitch and Stamer (2006) found that in Spanish, pictures with names from sparse neighborhoods were named more quickly than pictures with names from dense neighborhoods (i.e., the opposite of what was found in English; Vitevitch, 2002b). Similarly, the effect of PND in speech recognition was the opposite of that found in English. Using an auditory lexical decision task, Vitevitch and Rodríguez (2005) found that native Spanish speakers responded to Spanish words from sparse neighborhoods more slowly and less accurately than Spanish words from dense neighborhoods. Because English is less morphologically productive than Spanish, Vitevitch and Stamer (2006) hypothesized that the direction of PND effects may be dependent upon the influence of morphology in a given language (see also Arbesman, Strogatz and Vitevitch, 2010a).

The current tool, therefore, aims to provide language scientists, clinicians, and teachers with a valuable resource to further investigate the PND effect in another language; namely, Arabic [see Holliday, Turnbull and Eychenne (2017) for a recently developed database of PND statistics of Korean words]. Arabic is a Semitic language with a unique morphological structure (a detailed description of this structure is presented below). Therefore, studying PND effects in Arabic will provide valuable insight into the different ways PND affects spoken language processing in different languages, and has implications for the generality of models of speech production and spoken word recognition.

Several factors motivated us to develop a PND database for Arabic. First, Arabic is from the Semitic family of languages, whereas English (a Germanic language) and Spanish (a Romance language) are both Indo-European languages. Semitic languages, which also include Hebrew, are known for their nonconcatenative morphology. Nonconcatenative morphology differs greatly from the concatenative morphology used in English and Spanish, where a new lexical item is formed by putting together at least two distinct morphemes (e.g., run + ing = running).

Second, Arabic is the most widely spoken Semitic language, making it important to study and understand the cognitive machinery involved in the acquisition, perception, and production of this language. Furthermore, Arabic ranks fourth after Chinese (Mandarin), Spanish, and English in terms of number of first-language speakers in the world (Lewis, Simons, & Fennig, 2016). In terms of the number of countries where a language is the official language, Arabic ranks third behind English and French.

Third, when one considers the most commonly taught foreign languages, Arabic came second on the list of enrollments of the 15 most commonly taught languages in the United States between the years 1958 and 2016 (Modern Language Association report; Looney & Lusin, 2018). The enrollments in Arabic language classes increased 8568% in that time span.

Together these factors highlight why it is important for language scientists, clinicians, and teachers to consider PND effects in Arabic as a first language, or as a second/foreign language. These factors also highlight the potential impact of the present database. Below we discuss the special characteristics of the variety of Arabic (i.e., Modern Standard Arabic) that we used for the present database.

## Modern Standard Arabic (MSA)

Native Arabic speakers offer an interesting case of simultaneous use of two language varieties, namely MSA and a spoken language vernacular (SLV). Arabic SLVs differ among Arab countries, and sometimes different SLVs are spoken within the same country (e.g., Egypt and Saudi Arabia). In Arabic, this situation, termed *diglossia* (See Ferguson, 1959, for a discussion)*,* implies that MSA is used in written and formal spoken communications, and that the local dialect is used for more informal spoken interactions. However, the active mixing and interaction between Arabic varieties in the speech of native Arabic speakers is empirically attested, and suggests that spoken Arabic may be best described as a continuum of varieties rather than discrete ones (Parkinson, 2003).

MSA plays an important role in this continuum of dialects for a number of reasons. First, it represents the official language in the Arab world. Second, it is predominantly used in Arabic media in both written and spoken forms. Third, as Parkinson (2003) has noted, the usage of its verbal features increases with the increase in the speaker’s level of education, making it an important marker of self-identity. Finally, MSA has an important status in teaching Arabic as a foreign language. An observation made by Badawi (2006) indicates that in formal Arabic as a foreign language setting, MSA has been the variety taught most often. Others have even called for the teaching of MSA as a lingua franca for non-native speakers (Jaradat & Al-Khawaldeh, 2015). In the sections below, we present a detailed description of MSA phonological, morphological, and orthographic characteristics that are relevant to the creation of the current calculator.

## MSA phonemes

MSA has a relatively small inventory of vowels. It consists of only three short vowels /i/, /a/, and /u/ and their corresponding long ones /i:/, /a:/, and /u:/. Two more diphthongs complement the vowel sounds of MSA. These are created by combining the short vowel /a/ with the two glides /j/ and /w/ to produce the diphthongs /ai/ and /au/ found in English words such as *bite* and *bow*, respectively.

In contrast, MSA has a larger inventory of consonants, with 28 consonants. Unlike English, MSA has postvelar consonants. Namely, it has both uvular and pharyngeal consonants. In addition, MSA has four consonants that are characterized by emphasis; that is, producing the sound “with a raised and a tensed tongue” (Ryding, 2005, pg. 14) or retracting the tongue towards the pharyngeal wall (Amayreh & Dyson, 1998). These four emphatic consonants share the same International Phonetic Alphabet (IPA) symbols with their non-emphatic MSA consonants but with the *pharyngealized* IPA diacritic (ˁ) added (i.e., /tˁ/, /dˁ/, /sˁ/, and /ðˁ/). The 28 MSA consonants are shown in Table 1.

**Table 1:**
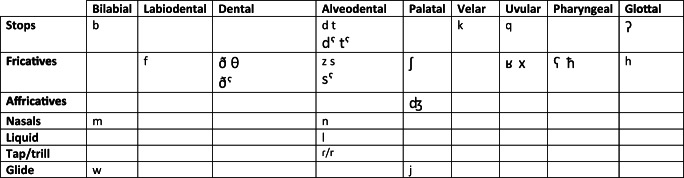
Consonants of Modern Standard Arabic

## Arabic morphology

In contrast to the concatenative morphology found in English, Spanish, and many other languages, Arabic uses nonconcatenative morphology to form words. For a word to be formed in Arabic, a root and a pattern must be mapped together (Holes, 1995). The root is a number of discontinuous consonants ranging from two to five, with roots containing three consonants being the most common. The pattern is basically a number of vowels (sometimes also containing other consonants) which work as a template for the root.

The root and pattern serve different functions. That is, whereas the pattern creates the phonological structure of the word and specifies its morphosyntactic properties, the root determines the semantic meaning of the word (Wright, 1995). The interaction between the root and pattern forms an Arabic word with complete phonological, morphosyntactic, and semantic properties. Table 2 contains examples of the mapping of the root {ktb} into different patterns.

**Table 2:** Mapping of the root {ktb} into different patterns

Pattern	Word	English Meaning
CaCaCa	Kataba (v)	He wrote
CaaCaCa	Kaataba (v)	He corresponded
CiCaaC	Kitaab (n)	book
CaaCiC	Kaatib (n)	writer
maCCaC	Maktab (n)	office
CuCuC	Kutub (n)	books
maCCuuC	Maktuub (pp)	written

v, verb; n, noun; pp, past participle

Empirical evidence suggests that the processing of roots and patterns in MSA is similar to the processing of roots and patterns in SLV (Boudelaa & Marslen-Wilson, 2013). Specifically, Boudelaa and Marslen-Wilson (2013) used auditory priming experiments to compare the procesing of roots and patterns in both MSA and the variety of spoken language vernacular known as Southern Tunisian Arabic (STA). They found that native Arabic speakers showed comparibly strong priming effects of roots in both varieties. The same was true for patterns. This was taken as evidence that MSA and STA “ …are cognitively processed and represented in fundamentally similar ways …” (Boudelaa & Marslen-Wilson, 2013, p. 1471). This finding suggests that native Arabic speakers possess comparible levels of morphological processing efficiency in both MSA and SLV, which means that any findings of PND effects in Arabic based on the present calculator in MSA are potentially generalizable to other Arabic varieties.

## Arabic writing system

The current PND calculator is based on a written database of MSA. Therefore, we describe in this section some important details regarding Arabic orthography to clarify some of the decisions we made in building the calculator. One important aspect of Arabic orthography is that it uses a combination of letters and diacritics (i.e., small marks used either under or above the letter representing the sounds they follow). Unlike all consonants and long vowels which are marked by letters, short vowels and gemination (consonant doubling or prolonging) are marked by diacritics. However, the use of diacritics is optional. Only when diacritics are used, such as in the Quran or in language teaching materials, can there be a close correspondence between sounds and writing symbols (i.e., letters or diacritics) rendering Arabic orthography shallow or phonemic. When they are not used, however, which is generally the case in normal writing, some Arabic words become homographic. Consider the undiacritized spelling of the Arabic word علم. Without diacritics, this word is homographic with other words that share the consonantal root {ʕlm} such as /ʕalima/ “he knew”, /ʕulima/ “it was known”, /ʕilm/ “Knowledge or science”, and /ʕalam/ “flag”. If diacritics are used, the word will be written (عَلِمَ), (عُلِمَ), (عِلْم) and (عَلَم), respectively. Skilled Arabic speakers overcome the problem of interpreting homographs by extracting the meaning from context.

Another interesting feature of Arabic orthography is that the letter *taa marbutah* (the “tied” *taa*) has different pronunciations in pausal and juncture contexts. This letter is either added to words word-finally to mark feminine gender in nouns or adjectives (e.g., /muʕallim/ “teacher” becomes /muʕallimah/ when in feminine form), or is intrinsically available in inherently feminine words (e.g., /madrasah/ “school”). The *taa marbutah* is pronounced as /h/ when in pausal form. On the other hand, it is pronounced as /t/ when the case-ending vowel attached to it is pronounced in juncture.

Fixed rules govern how some letters representing certain sounds can be spelled. For example, *hamza* (the symbol for the glottal stop sound) is spelled differently depending on its position in the word and the surrounding vowels (Ryding, 2005). The hamza can stand on its own, stand “on the line,” or “sit on a chair.” If a chair is required, it will take the shape of the letters representing long vowels in Arabic (ﻱ for /i:/, ا for /a:/, and ﻭ for /u:/). If the hamza stands on the line in a word such as ماء /ma:ʔ/ “water”, it will sit on a chair shaped like (ي) in ئةر /riʔah/ “lung”, shaped like (ا) in ألم /ʔalam/ “pain”, and shaped like (ﻭ) in لمؤم /muʔlim/ “painful”.

In all these examples, *hamza* is referred to as a *strong hamza* because it is part of the sounds in the word. However, when the hamza is not part of the sounds in the word but rather is added at the beginning of the word to help in the pronunciation of its initial consonant clusters, the hamza is considered to be weak. A weak hamza is only pronounced when the word is utterance-initial. It takes the symbol (ا) rather than the symbols (أ) or ( إ), which represent a strong hamza in initial position.

Similarly, the long vowel /a:/ in Arabic has more than one spelling variant. The derivational etymology of the word plays an important role in deciding the spelling variant used. For example, the words for “on” and “rise” in English are homophonic in Arabic (i.e., both pronounced as /ʕala:/), but they are spelled (ىعل) and ( اعل), respectively, where the underlined parts spell out the long vowel /a:/.

Finally, there are a few cases in Arabic where the same letter can spell out different sounds. For example, the letter yaa (ﻳ) can represent either the consonant /j/ or the long vowel /i:/. Similarly, the letter waw (ﻭ) can represent either the consonant /w/ or the long vowel /u:/.

## Method

### The corpus used in the Arabic Phonological Neighborhood Density Calculator

Given the characteristics of MSA described above, the corpus that would best underlie the Arabic Phonological Neighborhood Density Calculator (APNDC) should fit the following criteria. First, the corpus should contain words found in MSA as well as counts for how often the words occur in the language (either written or spoken). Ideally, the corpus should be large so that these word frequency counts are reliable. Second, the corpus should contain information regarding short vowels and gemination that are marked by diacritics (which are not typically used in normal writing in Arabic) to disambiguate potentially homophonous forms. Finally, the corpus should be current so that it accommodates the dynamic nature of MSA (i.e*.*, being the official language in the Arab world) in terms of the active and frequent addition of new words to keep up with advances in political, technical, and scientific fields.

Ar ten ten (Arts, Belinkov, Habash, Kilgarriff, & Suchomel, 2014) was the best fit for our criteria. It is a written corpus of Arabic gathered in 2012, comprising 5.8 billion words. A subset of Ar ten ten containing 115 million words, including diacritics, enabled us to disambiguate words. These diacritics were placed in the words after this subset of the corpus was processed with MADA (Habash, Rambow, & Roth, 2009). MADA is an Arabic language-processing tool that uses a morphological analyzer for MSA that disambiguates MSA words in context by reaching a preferred analysis for each undiacritized word. The 115-million-word subset, which was lemmatized and tagged for parts of speech by MADA, was loaded to a corpus manager called Sketch Engine (https://www. sketchengine.co.uk/).

Ar ten ten provides additional benefits. First, Ar ten ten included text from web domains in a variety of Arab countries providing a representative sample of the use of MSA in different geographical areas in the Arab world. Second, Ar ten ten adequately reflects the contextual usage of the words in the corpus because it only uses text from sentences. Third, Ar ten ten sampled text from social websites where users shared their complaints and concerns (e.g., http://humum.net), or religious and social questions (e.g., http://m.islamweb.net). Thus, the corpus contains MSA as used in various natural contexts rather than in fixed, scripted ones (e.g., news texts). Finally, Ar ten ten was also used to construct a web-based phonotactic probability calculator for Arabic (Aljasser & Vitevitch, 2018), providing a common dataset for the phonotactic probability calculator and the neighborhood density calculator described here.

A frequency wordlist containing the 100,000 most frequent MSA words was purchased from Sketch Engine (https://www. sketchengine.co.uk/). For regular words, the lemma (i.e*.,* the uninflected dictionary citation form of the word) was used for the database that underlies the APNDC. For example, when a word form with the feminine marker taa marbutah (e.g., معلمة /muʕallimah/) or the plural marker/u:n/ (e.g.,  /mu?minu:n/) appeared in the corpus, the lemmatized uninflected forms /muʕallima/ and /mu?min/ were used in the calculator. However, for irregular forms, such as irregular (broken) plural forms, the internal structure (i.e., the pattern) of the word was changed (e.g., providing the lemma /tˁa:lib/ ﻃﺎﻟﺐ for the broken plural /tˁulla:b/ ﻃﻼﺏ ). To avoid loss of the phonological information in the Arabic vowel patterns contained in these irregular forms, we instead used the phonemic transcriptions of the irregular forms.

The lemmas in the wordlist were analyzed in the fully vowelized Buckwalter (2002) transliteration. This transliteration represents Arabic script by using ASCII characters to romanize the orthographic forms of the words by reflecting the spelling variants in Arabic script of the same phonemes discussed above. Therefore, similar to Arabic orthography, Buckwalter transliteration uses several different orthographic characters for the glottal stop and the long /a:/ vowel. Because we were interested in phonological rather than orthographic representations, we encoded all different variants (transliterations) of the same sound (i.e., glottal stop and /a:/) as one symbol. Furthermore, to avoid Buckwalter’s (2002) dual function of symbols, we used the uppercase symbols I, A, and U to transcribe the long vowels / i:/, /a:/, and /u:/, respectively, and we used the symbols Y and W to transcribe the two diphthongs /ai/ and /au/ as is shown in Table 3.

**Table 3:**
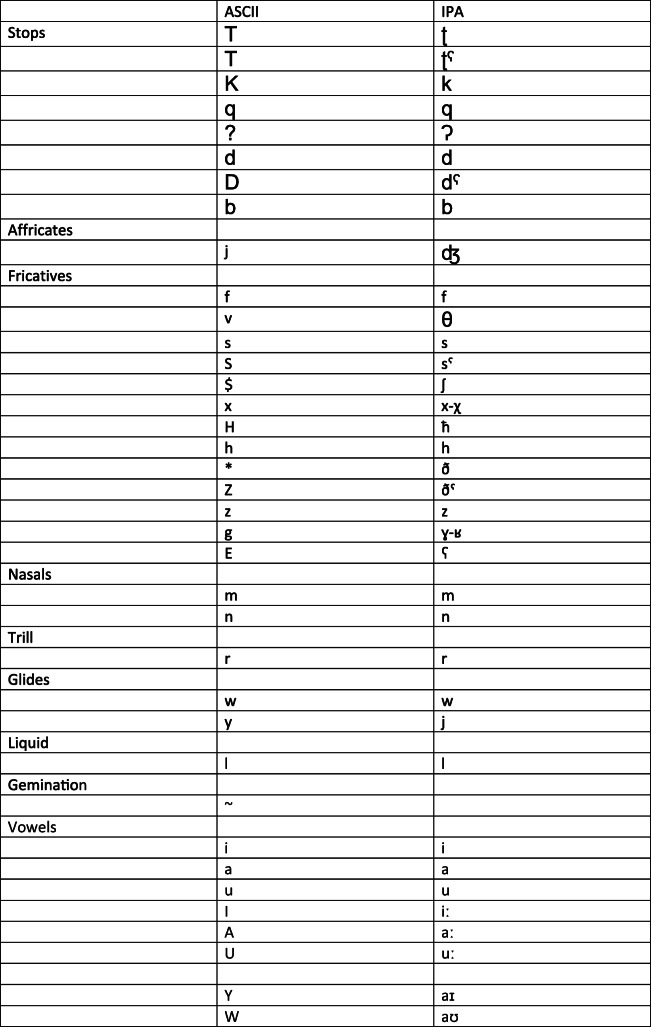
ASCII characters used in the APNDC and International Phonetic Alphabet (IPA) transcriptions

Most of the ASCII characters used in the calculator are based on Buckwalter’s (2002) transliterations, with necessary amendments to accommodate certain characteristics of the MSA phonemic inventory.

Additionally, other choices were made regarding which pronunciation to keep for some sounds. Recall that weak *hamza,* which represents the glottal stop, is only pronounced when the word is utterance-initial. We chose to omit it. Therefore, a word starting with a weak hamza, such as ابن, was transcribed as /ibn/ rather than /Ɂibn/. Also, the pronunciation /h/ was chosen over /t/ for taa marbutah, to preserve a more representative frequency of the sound /h/ in word-final position in MSA (e.g., /muʕallimah/).

Finally, the Ar ten ten corpus provided the same lemmas for all inflected forms of the same word. This resulted in a large number of homophones among the lemmas of the wordlist. Other homophonic words included those containing /a:/ as in the examples given above. For example, and as mentioned above, the words for “on” and “rise” in English are homophonic in Arabic (i.e., both pronounced as /ʕala:/), but they are spelled  and  respectively. Multiple forms of the homophones were reduced to the most frequent form and included in the calculator. In the present example, the frequency of the preposition  “on” is one of the highest in the corpus with 1,586,898 occurrences, whereas the frequency of  “rise” is only 2764. Therefore, the frequency value of the more frequent form was the one added to the calculator.

All proper nouns and directly borrowed words (except those with no Arabic equivalents) were also removed from the corpus. The pronunciations and transcription of all the entries in the database (lemmatized wordlist) were manually checked and edited (where needed) by a trained native speaker of Arabic (F.A.) with the help of the Almaany online Arabic dictionary https://www.almaany.com/. The final database had 11,164 unique lemmas of MSA in phonemic transcription. Frequency counts of these lemmas were used in the APNDC.

As described above, phonological similarity among words was assessed using a simple and widely-used method (Greenberg & Jenkins 1964, Landauer & Streeter 1973) based on the Hamming or Levenshtein distance (see Luce & Pisoni, 1998 for other ways to operationally define phonological similarity). Two words are considered phonologically similar if the addition, deletion, or substitution of a phoneme in any position of one word produces the other word. This method of defining phonological similarity is also used in the English and Spanish calculators (Vitevitch & Luce, 2004; Vitevitch, Stamer & Kieweg, 2012), allowing users to compare phonological density across languages.

### How to use the calculator

The APNDC can be freely accessed at https://calculator.ku.edu/density/about to calculate the neighborhood density of both real MSA words and pseudowords up to 15 phonemes in length. The gemination marker (~), which must be added after a geminated consonant, counts as a phoneme.

**Fig. 1:**
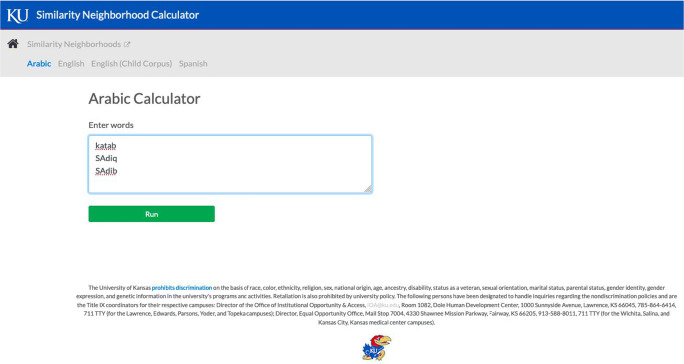
Depiction of the input field page for the Arabic Phonological Neighborhood Density Calculator. This is where the computer-readable phonemic transcription is entered.

The landing page offers the user the option to calculate neighborhood density for English, Spanish, or Arabic, and also has links to documents (similar to Table 3) that enable the user to convert IPA symbols to the computer-readable transcription used in the various calculators. After selecting “Arabic” from the list of languages, a screen like the one depicted in Fig. 1 will appear.

**Fig. 2:**
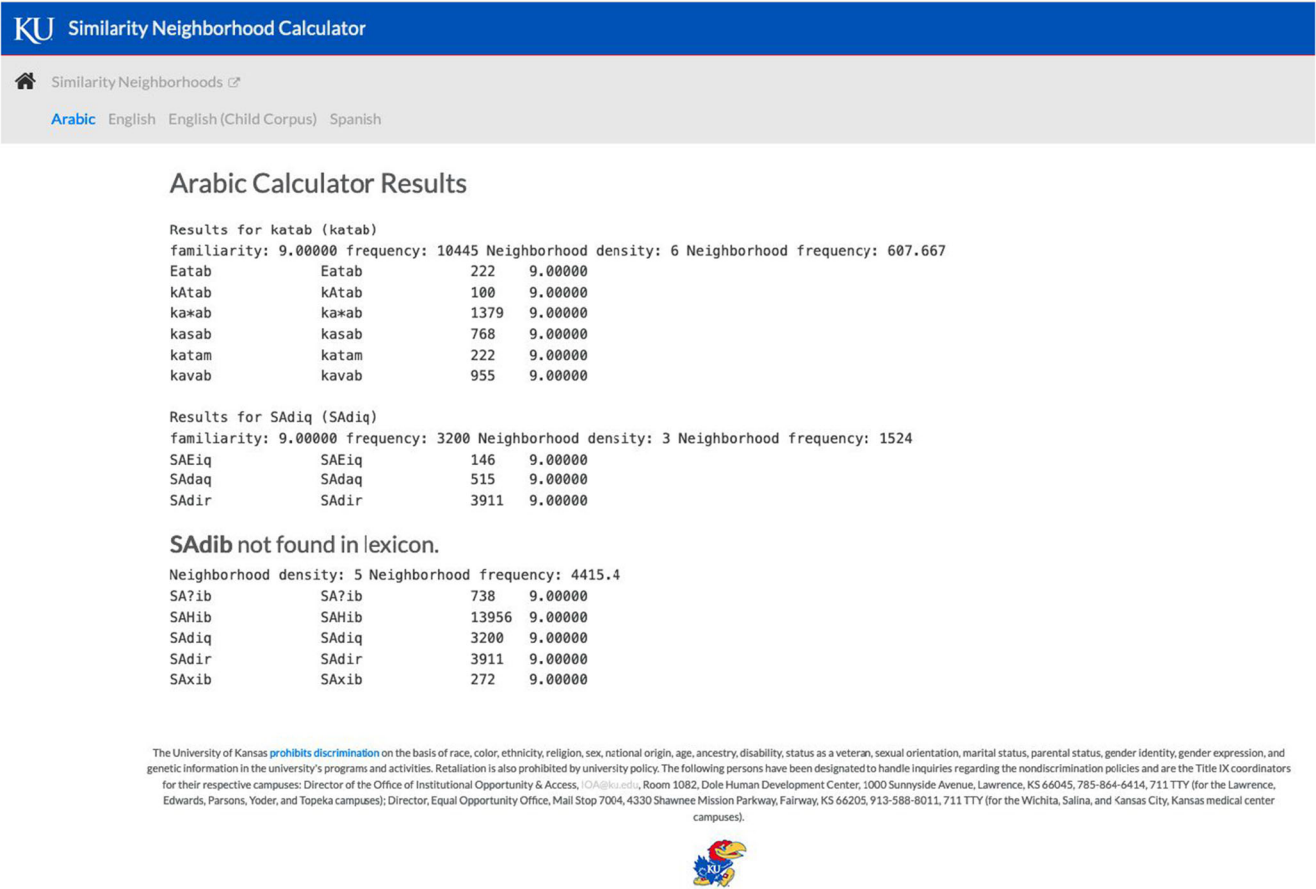
Output page for the Arabic word /katab/, the Arabic word /SAdiq/ “honest”, and the nonword /SAdib/

To calculate the neighborhood density of your items (words or nonwords), enter their phonemic transcriptions using the ASCII characters provided in Table 3 (and also available on the landing page for the APNDC). One item should be entered per line. This can be done either by typing the item directly into the field and using <Enter> to move to the next line, or by copying your items from a text file and pasting them into the field (one item per line, using a hard return). There is no limit to the number of items you can enter (by either typing or copying and pasting) in the calculator’s field, thus facilitating batch processing of many words. However, speed of calculation is affected by the number of items that have been entered into the input window, as well as by the amount of traffic on the network. In Fig. 1, the ASCII transcriptions of the Arabic word /katab/ “to write,” the Arabic word /sˁa:diq/ “honest”, and a nonword /sˁa:dib/ have been entered. By clicking the Run button, the neighborhood density of the items will be calculated and appear on a new page, as in Fig. 2.

For each item the word (or nonword) that was entered will appear, followed by summary information about the word (or nonword) and the list of phonological neighbors. As shown in Fig. 2, nonwords are clearly identified as “not found in the lexicon.” Although the neighborhood density calculators for the other languages available on this website report familiarity ratings for most words on a 7-point scale, we do not have familiarity ratings for Arabic words. The convention in the other neighborhood density calculators is to report a familiarity rating of 9 (i.e., a value outside the bounds of the 7-point scale) to indicate the absence of a familiarity rating in the database for that word.

The frequency of occurrence in the APNDC is the number of times the word occurred in the corpus of 115 million words in the Ar ten ten wordlist. The number of phonological neighbors (i.e., neighborhood density) is provided, followed by the mean frequency of occurrence of the phonological neighbors (i.e., neighborhood frequency). The words that are phonological neighbors and their corresponding frequency of occurrence are reported below the summary information for each word. The information in the window can then be highlighted, copied, and pasted to a separate document and saved in the user’s preferred file format for further processing. To refresh the input window, we recommend clicking on the Arabic label (to identify which neighborhood density calculator you wish to use) rather than using the back button on your browser. Using the back button on your browser will result in the word(s) or nonword(s) you previously entered appearing in the window, which will require you to select all, delete, and then enter or cut-and-paste a new set of entries.

## Conclusion

The Arabic Phonological Neighborhood Density Calculator described in the present work was based very closely on the Phonological Neighborhood Density Calculator for English (and Spanish) that is also available for use on this website. Like the English version, the APNDC uses a very simple and widely used measure of phonological similarity based on information theory, and that is relatively neutral regarding linguistic theory. Despite the simplicity of the one-phoneme metric, measuring phonological similarity and neighborhood density in this way has proven useful to language researchers from a variety of areas for stimulating new research questions and in examining a number of populations (e.g., Botezatu & Mirman, 2019; Chen, Vaid, Boas & Bortfeld, 2011; Farquharson, Centanni, Franzluebbers & Hogan, 2014; Goh, Suárez & Yap, 2009; Gordon & Kurczek, 2014; Munson & Solomon, 2004; Storkel, Maekawa & Hoover, 2010). We hope that the Arabic Phonological Neighborhood Density Calculator described here will similarly stimulate new research questions, and prove useful in the classroom and in the clinic as well.
